# Analytical Derivatives of Symmetry-Adapted Perturbation Theory Corrections for Interaction-Induced Properties

**DOI:** 10.1021/acs.jctc.5c00238

**Published:** 2025-04-25

**Authors:** Bartosz Tyrcha, Tarun Gupta, Konrad Patkowski, Piotr S. Żuchowski

**Affiliations:** † Institute of Physics, Faculty of Physics, Astronomy and Informatics, Nicolaus Copernicus University in Toruń, Grudziadzka 5/7, 87-100 Toruń, Poland; ‡ Department of Chemistry and Biochemistry, Auburn University, Auburn, Alabama 36849, United States

## Abstract

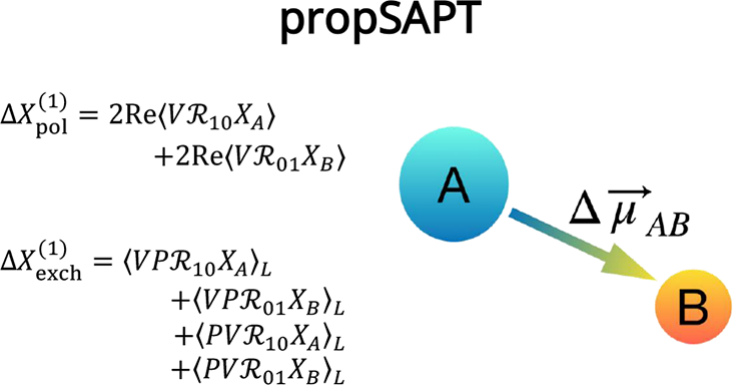

A new approach that allows for the calculation of interaction-induced properties exclusively from the properties of monomers is presented. The method is derived in the spirit of the symmetry-adapted perturbation theory (SAPT). The interaction-induced property is presented in the first order of the molecular interaction operator, including the exchange effects. Test calculations of the interaction-induced dipole moment were carried out for a number of small nonpolar and polar atomic and molecular dimers. The numerical results show that the analytical first-order corrections proposed in this paper reproduce the finite-field treatment of the first-order corrections of SAPT. Compared to supermolecular approaches, the performance of the finite-field SAPT (up to the second order) constitutes an insightful alternative for calculations of interaction-induced properties.

## 1 Introduction

Although noncovalent interactions are orders of magnitude weaker than covalent bonds, they critically affect many physical properties of chemical systems. They shape the structures of molecular crystals, influence the bulk properties of gases, affect the spectroscopy of molecular complexes, and alter chemical reactions. Beyond phenomena related to changes in the potential energy of weakly bound systems, these interactions can also lead to electron density deformations, causing electron flow between different parts of the interacting systems. Properties sensitive to these changes in electron density deserve special attention. For example, modifications to dipole moments induced by interactions, which determine collision-induced absorption coefficients^1−6^ depend, apart from potential energy surfaces, on dipole moment surfaces. Similarly, NMR spectroscopy parameters in crystals, such as shielding constants and chemical shifts, are affected by intermolecular forces.^7,8^ In addition, induced dipole moments and polarizabilities influence the dielectric properties of gases and their mixtures.^9−11^ More recently, scattering Feshbach resonances in the ultralow kinetic energy regime turned out to be driven by the modification of the hyperfine atomic coupling in collisions of alkali metal atoms with closed shell species.^12−14^

Among many approaches, symmetry-adapted perturbation theory (SAPT) has proven to be one of the best tools for studying noncovalent interactions. The current range of applicability of SAPT spans small^15,16^ to large systems with 100–200 atoms.^17−20^ In addition, the range of possible systems includes open-shell and multiconfigurational systems^21−23^ or even autoionizing dimers.^24−26^ The central concept of the SAPT methods is the partitioning of the dimer Hamiltonian into the Hamiltonians of the monomers and the interaction operator between the subsystems
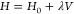
1and performing perturbation theory expansion in terms of λ with proper restoration of permutation symmetry of the dimer wave function.^27,28^ In numerous benchmark studies, SAPT demonstrated competitive accuracy with respect to gold-standard quantum chemistry methods.^29−31^

Traditionally, SAPT has been predominantly utilized for the analysis of interaction energies, establishing it as an essential tool for exploring multidimensional potential energy surfaces, conducting interaction energy decompositions, and developing force fields.^32−34^ However, its application has remained largely restricted to these areas, with limited use in investigating molecular properties or energy gradients. Given the multitude of challenges in chemical physics that critically rely on energy derivatives, there is a clear need for progress to expand SAPT’s scope into these new directions.

Despite this lack of theory for SAPT derivatives, finite-field numerical differentiation has enabled the study of some essential properties of van der Waals complexes. For example, Heijmen et al.^35^ demonstrated that the interaction-induced dipole moment of a simple complex such as He···H_2_ can be derived from the derivative of SAPT components with respect to an external electric field. In addition, several studies have focused on the electric polarizabilities and hyperpolarizabilities of complexes using SAPT or energy decomposition schemes.^10,36−39^ More recently, Iglesias-Reguant et al.^40,41^ presented an innovative approach to understand the infrared spectroscopy of molecular complexes through the decomposition of interaction energy and derivatives of its components with respect to normal modes.

As two molecules approach each other from infinite separation, their properties change because of the distortion of their electronic wave functions. Such change is referred to as the interaction-induced property and can be defined in a similar way as the interaction energy
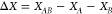
2where *X* denotes a given property that is measured (calculated) at the given geometry for the dimer complex and separate monomers. Interaction-induced properties (from now on, we will use the abbreviation IIP) have been studied for quite a long time, notably starting with Richard Feynman and Hans Hellmann independently in the late 1930s.^42−44^ Much later, Hirschfelder and Eliason^45^ performed a detailed analysis of the second-order corrections in the *V* operator for two hydrogen atoms. Buckingham^46^ studied polarizability and hyperpolarizability of a pair of atoms and used double perturbation theory for the leading asymptotic terms. In 1990, Hunt^47^ rigorously proved Feynman’s hypothesis on the behavior of dipole moments and derived closed formulas for the effects of polarization on molecular properties in terms of response functions in the asymptotic region. As we briefly stated before, one of the primary reasons for studying dipole moment surfaces is collision-induced absorption.^48,49^

In this paper, we consider only the first-order properties (i.e., linear with respect to the external field strength associated with *X*), which can be described by the Hellmann–Feynman theorem. To this end, we start with the approach introduced by the group of Jeziorski^50^ in which the IIP are computed directly, without the necessity of computing the dimer property, and adopt it to the SAPT formalism, as a series of perturbations in *X*. Then, we present a formulation for the single reference case, both for Hartree–Fock and Kohn–Sham formalisms. The formulation introduced by us is applicable to any interaction-induced first-order properties, but the specific numerical tests presented here pertain to the interaction-induced dipole moment. This quantity is particularly important to understand and interpret CIA data from laboratory measurements and astrophysical observations, and it is highly beneficial to leverage additional insights provided by physical decomposition.^1^ At the first-order level introduced here, the interaction-induced dipole moment is computed as a sum of electrostatic and exchange contributions, each of them further splitting into the interaction-induced response of molecule *A* and molecule *B*. We provide numerical tests of our approach against supermolecular calculations of the dipole moment at the CCSD(T) level and from finite-field SAPT. We use the abbreviation propSAPT for the new framework presented here.

## 2 Theory

From a formal perspective, the formulation of the IIP theory represents a double perturbation approach and is treated as such in this section. Historically, Hunt^47^ introduced the foundational theory of IIP in the asymptotic region,^47^ where the *V* operator is expanded into a series of multipoles. The theory presented here extends these calculations to include polarization terms in the valence-overlap region, where the multipole expansion fails to converge. In addition, it encompasses exchange interactions, broadening the model’s applicability beyond traditional asymptotic limits.

### 2.1 General Formulation of Theory of Interaction-Induced Properties

We begin with an overview of the theory presented by Piszczatowski, Łach, and Jeziorski,^50^ focusing specifically on one-electron properties. For a system comprising two subsystems, we assume that the Schrödinger equations for the monomers are solvable, that is, *h*_*A*_ϕ_*A*_ = *e*_*A*_ϕ_*A*_ and *h*_*B*_ϕ_*B*_ = *e*_*B*_ϕ_*B*_. When the interaction between subsystems *A* and *B* is introduced, we assume the combined system satisfies the Schrödinger equation for the dimer:
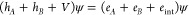
3where *V* represents the intermolecular interaction operator, and *e*_int_ is the interaction energy. At this stage, ψ denotes the dimer wave function, and its expansion is yet to be considered (vide infra).

Introducing a one-electron perturbation with the formal expansion parameter ζ*X* = ζ(*X*_*A*_ + *X*_*B*_), we will obtain perturbed analogs of interacting and noninteracting equations:
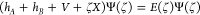
4
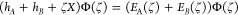
5The physical meaning of *E*_*A*_(ζ), *E*_*B*_(ζ), and *E*(ζ) is that they represent monomer and dimer energies perturbed by the *X* operator, respectively. By projecting the above equations on Φ(ζ), Piszczatowski et al.^50^ demonstrated that the perturbative effect of *X*_*A*_ + *X*_*B*_ on the difference energy of the dimer and monomers is given by
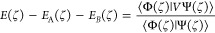
6Following the Hellmann–Feynman theorem, the interaction-induced expectation value of *X* can be derived by differentiating the above equation and setting ζ = 0, resulting in
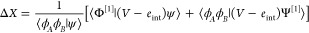
7where Ψ^[1]^ and Φ^[1]^ represent the first-order corrections (in ζ) to the wave functions Ψ and Φ. Note that we use [1] to indicate first-order with respect to the *X* operator. The induced property Δ*X*, introduced in eq 2, is of first order by definition as we have obtained it from the Hellmann–Feynman theorem, so for that particular quantity, we remove the [1] superscript. The functions Ψ^[1]^ and Φ^[1]^ are the solutions of the following linear equations:
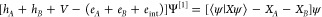
8and
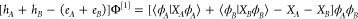
9The last equation is separable into *A* and *B* parts as
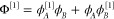
10where ϕ_*A*_^[1]^ and ϕ_*B*_^[1]^ are solutions of the following equations:
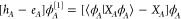
11
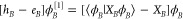
12

We next approximate the wave functions appearing in eq 7 using a double perturbation expansion of a dimer equation
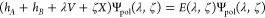
13with the exact solution in the double series form being
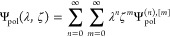
14where the Ψ_pol_^(*n*), [*m*]^ wave function is of *n*-th order in the interaction operator *V* and *m*-th order in the external perturbation *X*. It is worth noting that Ψ_pol_^(0), [1]^ = Φ^[1]^ and Ψ_pol_^(0), [0]^ = ϕ_*A*_ϕ_*B*_.

It is well-known that such a polarization wave function will not have a proper permutation symmetry. How can the exchange effects and the Pauli exclusion principle be incorporated into the presented theory? To restore the proper permutation symmetry, we use weak symmetry forcing, according to the procedure known from the symmetrized Rayleigh–Schrödinger (SRS) approach.^27,28^ It relies on projecting the polarization wave function onto the subspace of functions with correct symmetry by using the antisymmetrizer operator . Introducing it into the eq 7 yields
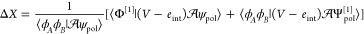
15where the functions Ψ_pol_^[1]^ and ψ_pol_ are obtained as the appropriate parts of the double perturbation expansion of eq 14, i.e.
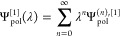
16and
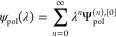
17

### 2.2 First-Order Interaction-Induced Property Corrections Based on Coupled-Perturbed Hartree–Fock Theory

In this part, we will consider the approximation to Δ*X* by employing the single Slater determinant wave function, corresponding to the same level approximation as in SAPT0. Such an approximation can smoothly be changed to the Kohn–Sham (KS) description, analogously to the SAPT(DFT) method.^51−54^ For convenience, let us use the same notation ϕ_*A*,*B*_ for the HF (or KS) Slater determinant as we used in the previous section. It is also helpful to introduce the normal-ordered form of spinorbital replacement operators^28,55,56^*a*_λ_^κ^ = *a*_κ_^†^*a*_λ_, and *a*_λλ′_^κκ^′^^ = *a*_κ_^†^*a*_κ′_^†^*a*_λ′_*a*_λ_ corresponding to monomer *A* and a similar set *b*_μ_^ν^, *b*_μμ′_^νν^′^^ corresponding to monomer *B*. The spinorbitals from now on will be labeled as α, β for occupied of *A* and *B* and ρ, σ for virtual of *A* and *B*, respectively. Moreover, κ, λ, μ, and ν will denote all molecular orbitals of either monomer, both occupied and virtual. Using this second-quantization language, the intermolecular interaction operator *V* reads as
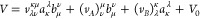
18where *v*_λν_^κμ^ = (κ(*r⃗*_1_)λ(*r⃗*_1_)|*r*_12_^–1^|μ(*r⃗*_2_)ν(*r⃗*_2_)) is a standard two-electron repulsion integral (ERI), (*v*_*A*_)_ν_^μ^ and (*v*_*B*_)_λ_^κ^ describe the electrostatic interaction with the nuclei of monomer *A* and *B*, respectively, and *V*_0_ is the nuclei–nuclei repulsion term. We also introduce a short-hand notation for scalar products:
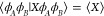
19
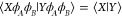
20

To proceed further, let us discuss the symmetry-forcing procedure in more detail. In many-electron theories, the symmetry forcing operator is the antisymmetrizer of all electrons in the system, denoted as . According to Moszyński (1994), this operator can be expressed in terms of the antisymmetrizers of subsystems:
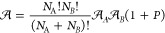
21where *P* includes all possible permutations interchanging electrons between two interacting subsystems. Using the second quantization approach, the intersystem permutation operator can be written as *P* = *P*_2_ + *P*_4_ + ··· where the operator of intermolecular exchange of *n* electron pairs^57^ in the second quantization method reads
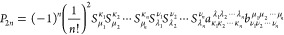
22When the above expansion is inserted into eq 15, in the first order of *V*, one obtains the first-order correction as
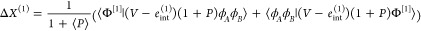
23

It is also important to specify the resolvent operator in this context. Here, it is understood as
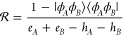
24The resolvent operator can be decomposed into a series of  operators corresponding to *n*-tuple excitations on *A* monomer and *m*-tuple excitations on *B* monomer
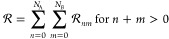
25where the  operator is defined as
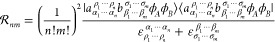
26and ε_ρ_1_···ρ_*n*__^α_1_···α_*n*_^, ε_σ_1_···σ_*m*__^β_1_···β_*m*_^ denote the differences of occupied and virtual spinorbital energies
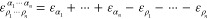
27Since *X* is a one-particle operator, it is straightforward to obtain that  will reduce to the sum of only two terms,  and . Furthermore, they read
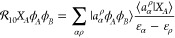
28
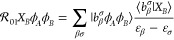
29which are solutions of eqs 11 and 12 when *h*_*A*_ and *h*_*B*_ are approximated by the Fock operators *f*_*A*_ and *f*_*B*_, and *e*_*A*_, *e*_*B*_ accordingly are sums of occupied spinorbital energies ε_α_ and ε_β_, respectively.

Equation 23 can be decomposed into polarization and exchange parts. The polarization correction can be obtained by neglecting all the intermonomer electron permutations arising from the *P* operator from the eq 23. Thus, we obtain
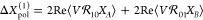
30In order to find the first-order exchange counterpart, it is enough to use the formula
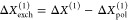
31and subtract eq 30 from eq 23. This leads to
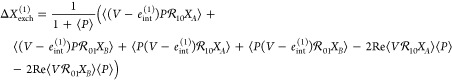
32Recently, in ref (57), two of us have demonstrated using the second quantization approach that unlinked terms appearing in the numerator cancel out with the ⟨*P*⟩ terms from the denominator (which is apparent after multiplying both sides of eq 32 by 1 + ⟨*P*⟩, see also ref (55)). Such a trick also allows the inclusion of *P*_2*n*_ operators of any order, allowing to go beyond the *S*^2^ approximation. Thus, the final formula for Δ*X*_exch_^(1)^ reads
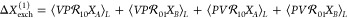
33where subscript *L* denotes that only the linked part of the expression is taken, i.e., only the terms for which the corresponding diagrammatic Goldstone-type representation constitutes a connected graph. This approach simplifies the notation strongly by omitting the so-called renormalization terms, whose only purpose is to cancel out unlinked diagram contributions of the main terms, similarly as in the case of first-order interaction energy exchange correction *E*_exch_^(10)^ in the single-exchange approximation *E*_exch_^(10)^(*S*^2^) = ⟨*VP*_2_⟩–⟨*V* ⟩⟨*P*_2_⟩ = ⟨*VP*_2_⟩_*L*_.^58,59^ It is also worth noting that a similar equation for Δ*X*_pol_^(1)^ was obtained for the change of a property in an external electric field by Błasiak in ref (60). Equations 30 and 33 constitute the main result of the present paper.

In order to include proper orbital relaxation effects on the first-order IIP corrections, the resolvent operators  and  in eqs 30 and 33 can be replaced respectively by the operators  and  that generate the solutions of the coupled-perturbed Hartree–Fock (CPHF) or coupled-perturbed Kohn–Sham (CPKS) equations,
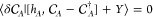
34
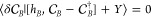
35where ,  are arbitrary single excitation operators, and ,  denote the solution of CPHF (or CPKS) equations with the perturbation *Y* (which will be specified below). Similar reasoning was applied to the second-order induction energy.^61^ It is well-known that ,  correspond to a response at the mean-field level without the explicit contribution of the dynamic correlation.

To obtain the formulas that are exact with respect to intermonomer permutations of electrons (thus, not involving any single, double, ... exchange approximation), we have used a similar approach as in the expressions for the exchange-induction energy presented in the ref (57), i.e., considering a series expansion into powers of overlap integral matrix elements *S* and performing its analytical summation as for a geometric series.

Equations 36 and 39 below present the resulting working formulas expressed in terms of molecular orbitals. These expressions assume real orbitals and the closed-shell restricted Hartree–Fock (RHF) (or restricted Kohn–Sham (RKS)) reference. The labels *a*, (*b*) refer to occupied orbitals, while *r*, (*s*) to virtual orbitals of monomer *A*, (*B*). Summation over the repeated indices is implied in all expressions. The first-order polarization correction to the IIP reads
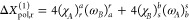
36where (χ_*A*_)_*r*_^*a*^, (χ_*B*_)_*s*_^*b*^ are the solutions of the CPHF (or CPKS) equations with the external perturbation being *X*_*A*_, *X*_*B*_ operators, respectively, and (ω_*A*_)_*b*_^*s*^, (ω_*B*_)_*a*_^*r*^ are the matrix elements of the electrostatic potential operators of unperturbed monomers and can be written as
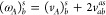
37
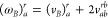
38The corresponding first-order exchange counterpart is
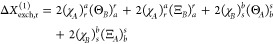
39The intermediates (Θ_*B*_)_*a*_^*r*^ and (Ξ_*B*_)_*a*_^*r*^ are defined as follows
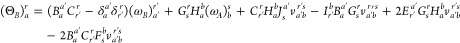
40and
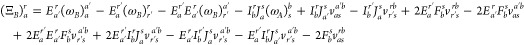
41The different possibilities of contracted strings of *S* overlap matrices appearing in the intermediates are
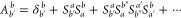
42
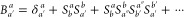
43
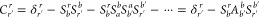
44
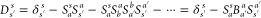
45
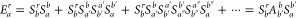
46
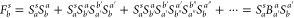
47
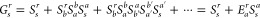
48
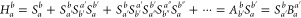
49
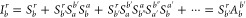
50
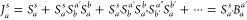
51The values of series *A*_*b′*_^*b*^ and *B*_*a′*_^*a*^ can be evaluated analytically as it was described by some of us in ref (57). Then, the remaining series of contracted *S* matrices defined in eqs 44–51 can be expressed through the analytically evaluated quantities.

Perhaps it is worth mentioning that the expression presented in eq 36 is somewhat similar to the orbital expression describing the second-order induction correction from SRS theory, precisely *E*_ind,r_^(20)^ = 2(*t*_*A*_)_*r*_^*a*^(ω_*B*_)_*a*_^*r*^ + 2(*t*_*B*_)_*s*_^*b*^(ω_*A*_)_*b*_^*s*^, where (*t*_*A*_)_*r*_^*a*^ and (*t*_*B*_)_*s*_^*b*^ amplitudes are obtained as solutions of CPHF equations with perturbations given by (ω_*B*_)_*a*_^*r*^ an (ω_*A*_)_*b*_^*s*^, respectively.^59^

Note that eqs 36 and 39 define the decomposition of the property change into physically meaningful components: polarization-driven and exchange-driven, respectively. Each of these terms also splits into change of the property on monomer *A* and *B* separately.

## 3 Details of Implementation

In order to derive the orbital formulas for first-order corrections to the induced quantity Δ*X*, the second quantization approach was used, and the final equations were implemented in Python utilizing the approach based on the Psi4NumPy^62^ framework – an interactive quantum-chemical environment designed for method development, rapid prototyping, and educational purposes. The basis for the newly developed expressions is the open-source quantum-chemical code Psi4.^63^ It is used to perform integral and SCF calculations and solve the CPHF and CPKS equations. All the necessary quantities are then exported from Psi4 into NumPy arrays, and their contractions are optimized and performed using the opt-einsum module.^64^

The first-order corrections to the induced property have been implemented using molecular orbitals. The contractions of corresponding orbital expressions have a numerical cost that scales as *N*^4^, where *N* is the number of molecular orbitals (due to the cost of the (Ξ_*B*_)_*a*_^*r*^ intermediate calculations). However, the cost of necessary CPHF (or CPKS) calculations scales as *N*^6^, and therefore, it constitutes the actual performance limiting factor of the proposed theory. Additionally, the density-fitting approximation may be employed to reduce the scaling of the propSAPT theory to *N*^3^ and the CPHF (or CPKS) calculations to *N*^5^.

The supermolecular Hartree–Fock and DFT calculations were carried out using the Psi4 code. In contrast, the reference finite-field SAPT and CCSD(T) calculations were performed using the Molpro^65^ program, employing the four-point stencil for the derivative calculations. The convergence threshold for the energy in the finite field calculations was set to 10^–11^*E*_h_.

The finite-field SAPT calculations were carried out for the Hartree–Fock and Kohn–Sham references. In both cases, they include corrections up to the second order of the interaction operator with the dispersion term calculated at the CPHF or CPKS level, respectively. Therefore, the HF-based SAPT calculations used in this work differ from the typically used SAPT0 level by the type of dispersion correction used.

For all systems and types of calculations, the dimer-centered aug-cc-pVTZ basis set^66−70^ was used. The propSAPT calculations of first-order interaction-induced corrections were carried out using the density-fitting approximation. For this purpose the aug-cc-pVTZ-RI basis^71^ was used as an auxiliary basis set, with the exception of calculations of He···Ne system where the aug-cc-pV5Z-RI basis^72^ was used. Additionally, CPKS calculations needed for the DFT-based propSAPT corrections utilize the adiabatic local-density approximation (ALDA)^73^ for the exchange-correlation kernel.

For the SAPT(DFT) and propSAPT(DFT) calculations, the PBE0 functional^74−76^ was used together with the gradient regulated asymptotic correction (GRAC)^77^ in order to restore the correct asymptotic behavior of the energy functional. The PBE0 functional was also used for the supermolecular DFT calculations. In this case, the asymptotic correction was not utilized.

## 4 Numerical Results

The numerical results from the theory presented in this work focus on the interaction-induced dipole moment in van der Waals complexes as an example of a one-electron IIP. The selection of test systems covers atom···atom, nonpolar, and polar complexes of high importance in molecular studies. These include: He···H_2_, CO_2_···N_2_, CO_2_···CH_4_, Ar···BF_3_, He···Ne, He···Be, H_2_O···Ar and H_2_O···H_2_O. The values of the induced dipole moment vector Δμ⃗ components in this work are presented as functions of the intermolecular displacement vector *R⃗*, pointing from the center of mass of monomer A to the center of mass of monomer B (for a system labeled as A···B). For the reference finite-filed calculations the field value was set to 0.001 *E*_h_/*ea*_0_ for all test systems, except the He···Ne where the field value of 0.005 *E*_h_/*ea*_0_ was used.

The dipole moment surfaces for He···H_2_, CO_2_···N_2_, and CO_2_···CH_4_ are crucial in calculating collision-induced absorption (CIA) coefficients, with applications to modeling the planetary atmospheres or interstellar clouds. Although the He···H_2_ complex has been very intensely studied,^35,78−83^ the latter systems are rather complex and to the best of our knowledge, no dipole moment surface calculations have yet been done for them. The selection of the Ar···BF_3_ complex was dictated by the fact that the B–F bonds are strongly polar. For this system, the induced dipole was also studied by Fowler and Stone.^84^ All geometries studied in this work are available in the Supporting Information.

Except the cases containing the water molecule, namely H_2_O···Ar and H_2_O···H_2_O, no molecule investigated here has a permanent dipole moment. However, at least one molecule in each non atom···atom complex has a permanent quadrupole moment. Thus, the induced dipole moment asymptotically decays for those systems like *R*^–4^, the electric field from a quadrupole, which originates from the first-order interaction in the operator *V*.^35,47^ Hence, the theory introduced here should be asymptotically correct and accurate for such a class of systems, provided that the monomer density is reproduced correctly.

Concerning the atom···atom cases, we investigated the He···Ne and He···Be systems, in which the induced dipole at long-range originates from dispersion interactions and thus vanishes as *R*^–7^.^42,43,47^ The reason to study these systems is to shed some light on the long-range behavior of the induced dipole moment in the first order compared to a dispersion-dominated behavior.

The last, but not least, subgroup of our test systems represents the case of polar molecules. Two systems were selected: H_2_O···Ar, constituting a model polar···atom example, and a water dimer, being a crucial polar···polar case. Selection of the H_2_O···Ar complex was additionally dictated by the fact that CIA coefficients for this system were not intensively studied yet.^1^ In the case of the water···water system, fast and reliable methods for induced dipole surface calculations may prove very useful in ab initio studies of the infrared and Raman spectra.^85^

**Figure 1:**
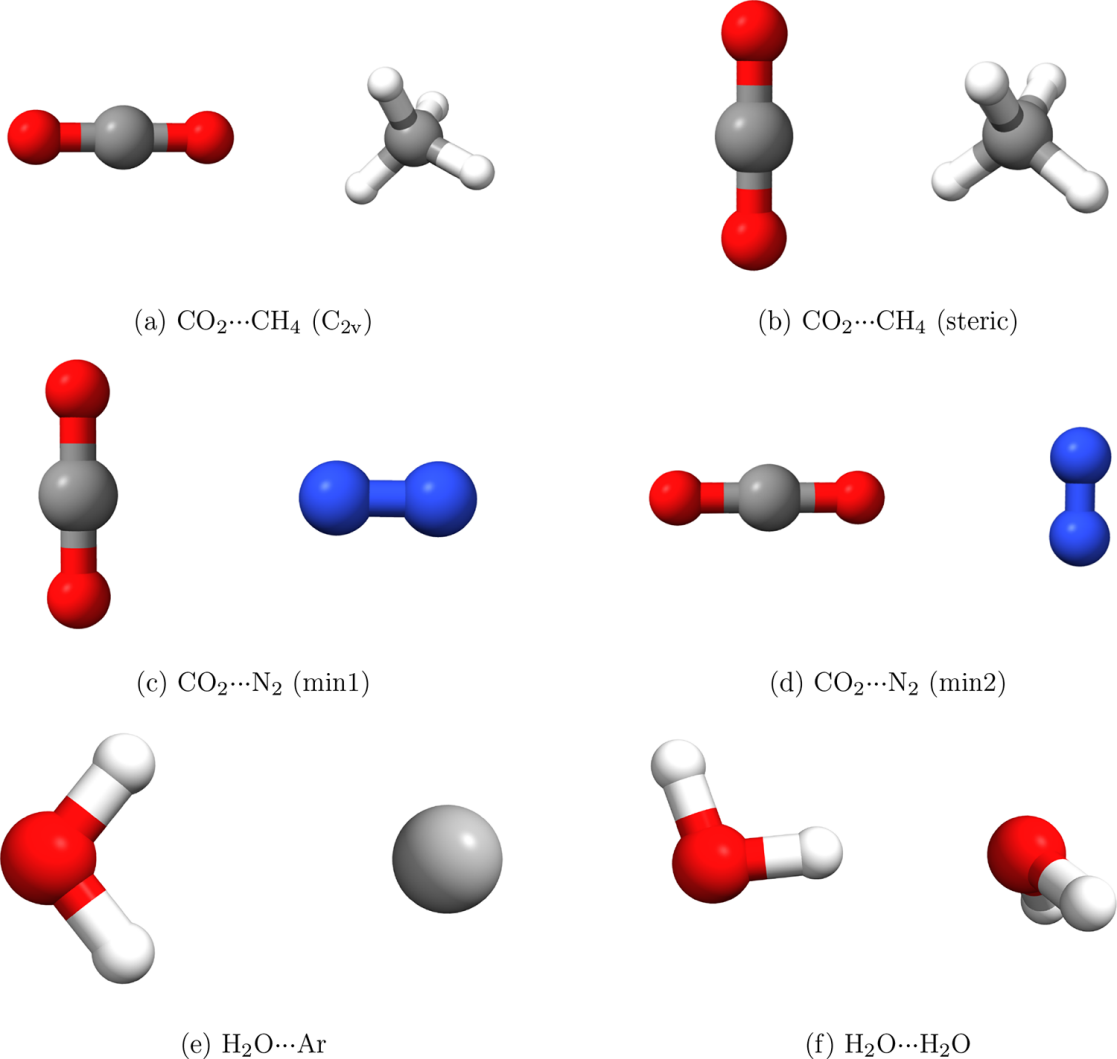
Spatial configurations of selected test systems. For *R*-dependent geometry scans, we used fixed molecular orientations in Jacobi coordinates, corresponding to stretching the vector connecting the centers of mass of the monomers.

The spatial configurations of some test systems used in this work are presented in Figure 1. Distance-dependent scans of the interaction-induced dipole moment were calculated for most systems. These scans were carried out using fixed molecular orientations, i.e., corresponding only to stretching the intermolecular displacement vector *R⃗*.

Table 1 collects essential comparisons of the propSAPT results with finite field calculations based on supermolecular methods (HF, DFT with PBE0 functional, and CCSD(T)) and first- and second-order SAPT with the coupled dispersion energy. Table 2 collects the individual corrections calculated with the HF and Kohn–Sham references (the latter using the asymptotically corrected PBE0 functional), along with the finite-field SAPT(HF) and SAPT(DFT) calculations (with the ALDA kernel in the latter case). Note that we expect the HF-based propSAPT results to be in precise agreement with the numerical derivatives of the first-order SAPT corrections, polarization (Δμ_pol,r_^(10)^) and exchange (Δμ_exch,r_^(10)^). The corresponding KS-based examples are not expected to have such a level of agreement due to the utilization of the ALDA kernel approximation in the CPKS calculations necessary for the propSAPT(DFT) theory.

**Table 1:** Values of the Interaction-Induced Dipole Moment Component along the Intermolecular Axis Specified by the Vector *R⃗* (Pointing from the Center of Mass of Monomer A to the Center of Mass of Monomer B for a System Denoted as A···B)^a^

system	*R* [*a*_0_]	propSAPT		FF-SAPT		supermolecular		
		HF	DFT	HF	DFT	HF	DFT	CCSD(T)
He···H_2_	5.00	–16.19	–17.52	–15.12	–16.31	–14.49	–13.42	–12.87
(linear)	7.00	–1.34	–1.38	–1.17	–1.20	–1.33	–0.81	–1.08
Ar···BF_3_	5.82	–71.69	–63.49	–88.64	–79.78	–87.83	–91.70	–88.82
	8.73	–18.21	–16.23	–19.23	–17.17	–19.00	–17.97	–17.89
CO_2_···N_2_	6.00	78.73	69.66	98.97	89.55	92.31	86.34	88.40
(min1)	10.00	12.16	11.45	12.69	11.95	12.55	12.11	12.14
CO_2_···N_2_	6.00	–144.81	–115.99	–194.90	–170.50	–173.21	–170.85	–162.35
(min2)	10.00	–14.50	–14.24	–15.20	–14.98	–15.25	–15.00	–15.31
CO_2_···CH_4_	6.00	–260.87	–238.55	–332.55	–307.74	–267.25	–211.41	–230.37
(C_2v_)	10.00	–17.05	–16.38	–17.44	–16.13	–18.01	–15.56	–15.00
CO_2_···CH_4_	6.00	37.98	25.32	51.83	42.08	46.61	46.79	45.05
(steric)	10.00	8.92	8.07	9.45	8.54	9.18	8.52	8.25
He···Ne	5.09	–0.15	–0.47	–0.12	–0.32	0.01	–0.17	–0.33
	5.65	–0.06	–0.19	–0.06	–0.15	–0.01	–0.05	–0.16
He···Be	6.37	–60.44	–49.55	–54.99	–46.46	–44.25	–27.43	–28.54
	7.96	–9.62	–6.83	–7.60	–4.88	–7.30	–1.48	–3.43
	9.95	–0.82	–0.48	–0.38	–0.06	–0.64	0.69	–0.05
H2O···Ar	5.00	88.82	95.79	104.04	113.01	98.95	113.23	109.88
	7.00	41.19	41.29	44.02	43.92	44.13	45.94	43.89
H_2_O···H_2_O	3.04	261.59	254.18	322.77	317.41	356.56	385.35	371.42
	3.80	166.26	166.96	193.95	197.66	205.65	231.28	218.72
	4.75	100.38	103.31	110.95	116.04	113.64	129.31	122.00

a All values of dipole moment are given in 10^–3^*ea*_0_.

**Table 2:** Decomposition of the First-Order Interaction-Induced Dipole Moment Components along the Intermolecular Axis into Polarization (Δμ_pol,r_) and Exchange Parts (Δμ_exch,r_)^a^

system	*R* [*a*_0_]	propSAPT		propSAPT(DFT)		FF-SAPT		FF-SAPT(DFT)	
		Δμ_pol,r_^(10)^	Δμ_exch,r_^(10)^	Δμ_pol,r_^(1)^	Δμ_exch,r_^(1)^	Δμ_pol,r_^(10)^	Δμ_exch,r_^(10)^	Δμ_pol,r_^(1)^	Δμ_exch,r_^(1)^
He···H_2_	5.00	–0.37	–15.82	0.10	–17.63	–0.37	–15.82	–0.08	–17.42
(linear)	7.00	–0.81	–0.53	–0.77	–0.61	–0.81	–0.53	–0.83	–0.57
Ar···BF_3_	5.82	–96.19	24.50	–85.76	22.27	–96.19	24.50	–85.84	22.34
	8.73	–18.44	0.23	–16.46	0.23	–18.44	0.23	–16.42	0.23
CO_2_···N_2_	6.00	107.00	–28.27	103.97	–34.31	107.00	–28.26	103.18	–33.80
(min1)	10.00	12.20	–0.03	11.50	–0.06	12.20	–0.03	11.43	–0.05
CO_2_···N_2_	6.00	–81.75	–63.06	–110.37	–5.62	–81.76	–63.06	–106.54	–15.26
(min2)	10.00	–14.15	–0.34	–13.99	–0.25	–14.16	–0.34	–13.92	–0.25
CO_2_···CH_4_	6.00	75.72	–336.59	94.14	–332.70	75.72	–336.59	87.41	–325.24
(C_2v_)	10.00	–15.62	–1.43	–13.74	–2.64	–15.62	–1.43	–13.46	–2.34
CO_2_···CH_4_	6.00	78.77	–40.80	82.53	–57.21	78.77	–40.79	78.07	–49.13
(steric)	10.00	8.99	–0.07	8.24	–0.17	8.99	–0.07	8.06	–0.16
He···Ne	5.09	–0.11	–0.04	–0.03	–0.44	–0.11	–0.04	–0.05	–0.31
	5.65	–0.01	–0.05	0.01	–0.19	–0.01	–0.05	–0.02	–0.12
He···Be	6.37	15.27	–75.71	12.53	–62.08	15.26	–75.70	13.14	–65.26
	7.96	2.31	–11.93	1.60	–8.44	2.30	–11.92	1.58	–8.42
	9.95	0.18	–1.00	0.11	–0.58	0.18	–1.00	0.10	–0.57
H_2_O···Ar	5.00	61.54	27.28	40.82	54.97	61.54	27.28	40.56	55.90
	7.00	40.05	1.14	37.88	3.41	40.05	1.14	37.91	3.38
H_2_O···H_2_O	3.04	223.45	38.14	225.13	29.05	223.45	38.14	226.31	26.42
	3.80	166.66	–0.40	172.04	–5.07	166.66	–0.40	172.93	–6.73
	4.75	103.42	–3.04	108.24	–4.93	103.42	–3.04	108.57	–5.27

a All values of dipole moment are given in 10^–3^*ea*_0_. Note that the propSAPT and FF-SAPT columns should match exactly. propSAPT and FF-SAPT(DFT) values are, to some extent, expected to differ due to the ALDA kernel approximation.

### 4.1 He···H_2_

**Figure 2:**
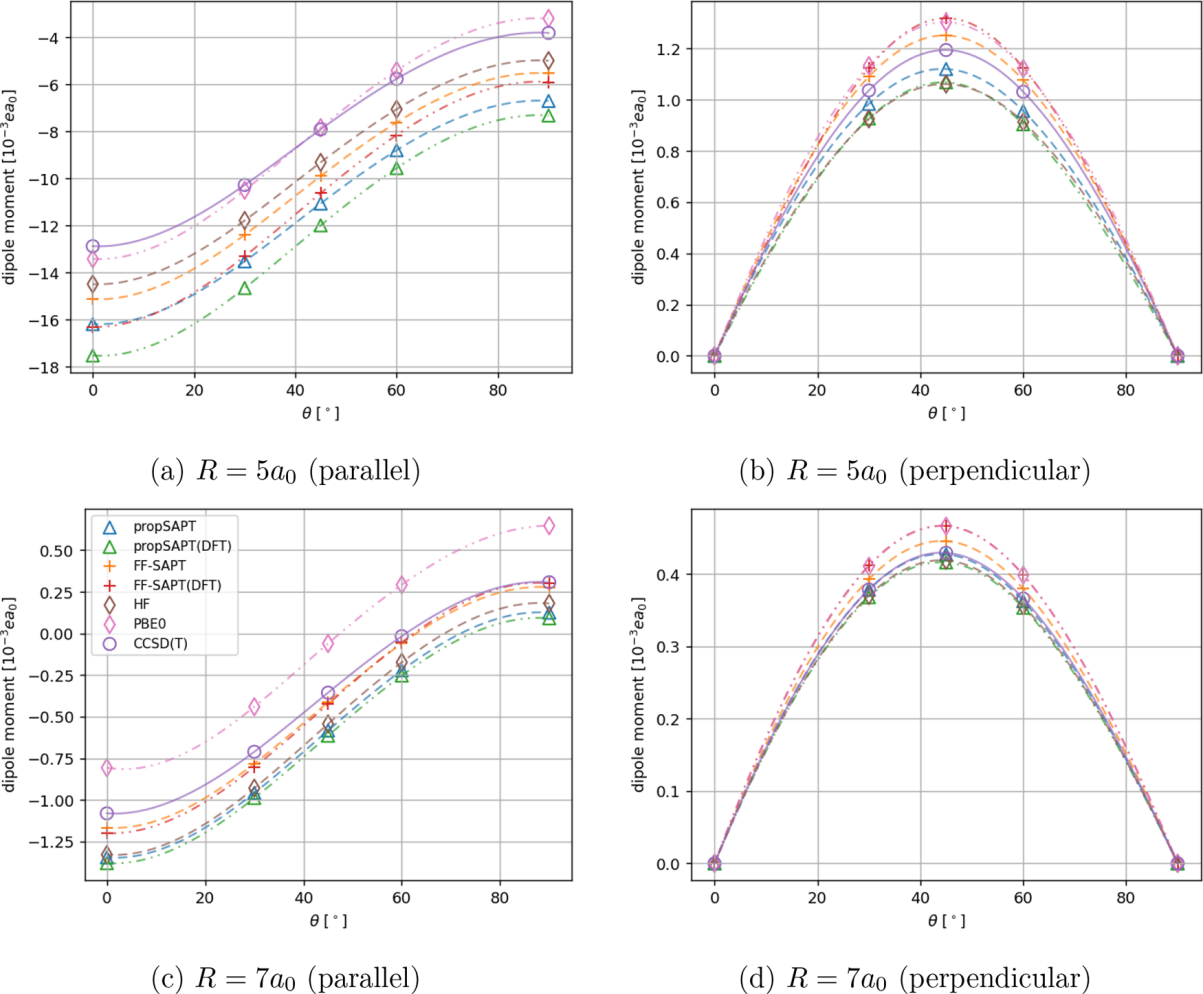
Angle-dependent comparison of interaction-induced dipole moment for the He···H_2_ system for two intermolecular separations calculated using the propSAPT approach, finite-field SAPT to the second order (FF-SAPT), and supermolecular methods. The parallel and perpendicular components with respect to the intermolecular axis are presented.

**Figure 3:**
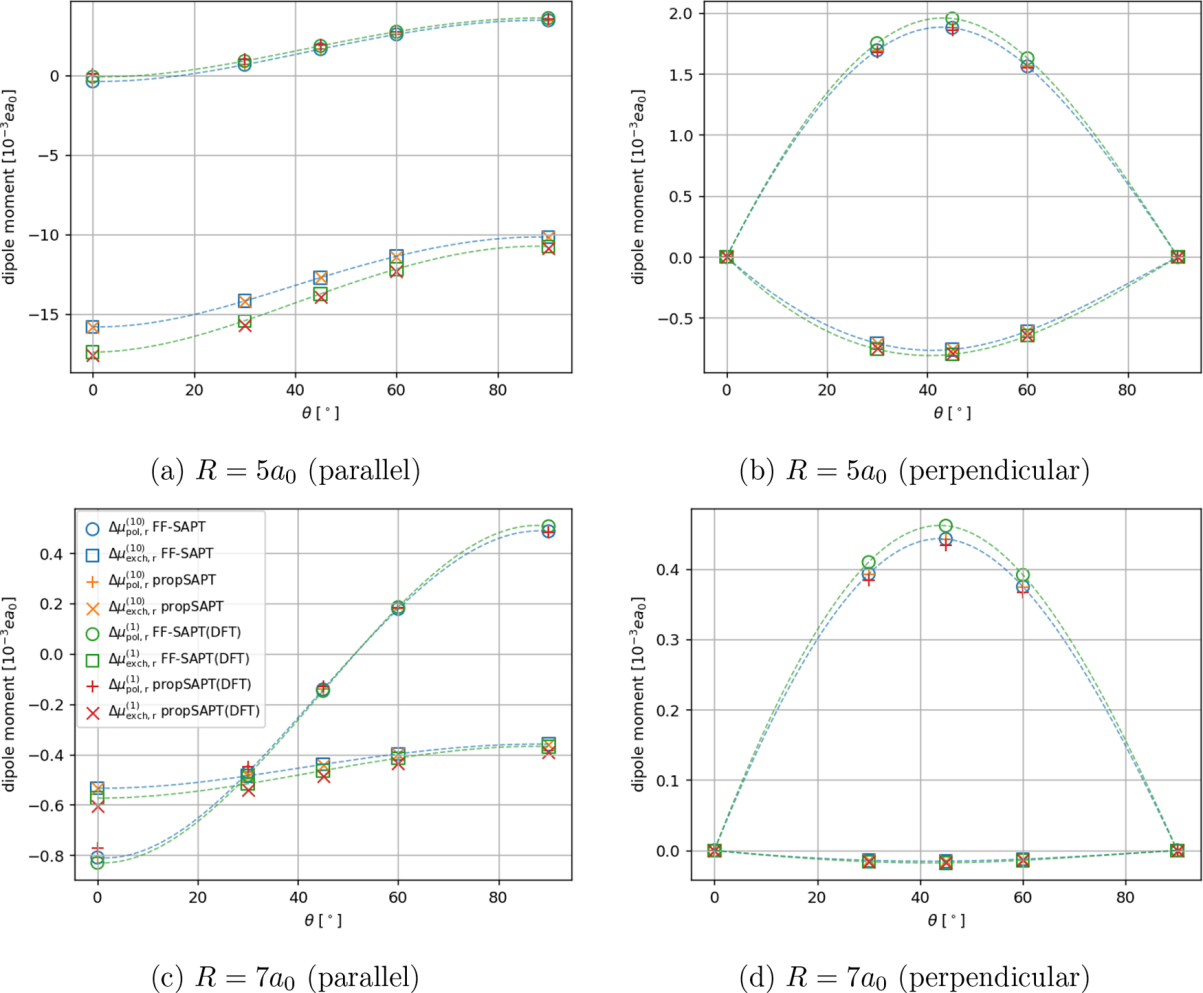
Angle-dependent comparison of the first-order polarization and exchange contributions to the interaction-induced dipole moment vector for the He···H_2_ system for two intermolecular separations. The parallel and perpendicular components with respect to the intermolecular axis are presented. Note that for the Hartree–Fock-based propSAPT contributions (orange plus and cross markers), we expect exact matching with the finite-field numerical derivatives of SAPT (blue box and circle markers).

We begin by discussing the interaction induced dipole moment of the He···H_2_ system. Following the approach of Heijmen et al.,^35^ we examined the same geometries: the angular dependence of the interaction-induced dipole moment for the angle between the molecular axis and the line connecting the centers of masses ranging from 0° to 90°, and its two components: perpendicular and parallel to the intermolecular axis, at distances of 5*a*_0_ (which corresponds to the van der Waals minimum region) and 7*a*_0_, respectively. Figures 2 and 3 present the angular dependence of the induced dipole compared with the finite-field (FF) methods and its decomposition into the polarization and exchange parts. Interestingly, the induced dipole moment is relatively insensitive to electronic correlation effects. Even the HF description alone provides a reasonably good approximation, overestimating the CCSD(T) results by 23% at 7*a*_0_ and 13% at 5*a*_0_ for the linear geometry presented in Table 1. The supermolecular DFT calculations agree with CCSD(T) quite well for 5*a*_0_, but as the atom···molecule separation increases, supermolecular DFT starts to deviate from CCSD(T) quite significantly.

The interaction-induced dipole moments calculated with propSAPT overestimate the CCSD(T) results by 26% for HF orbitals and 36% for PBE0 Kohn–Sham orbitals, respectively, at the distance of 5*a*_0_. For 7*a*_0_, the accuracy of propSAPT slightly improves to 24 and 28%, respectively. We do expect the propSAPT results to be approximate since they include only a first-order correction. However, the amount of second- and higher-order behavior is relatively small and positive, which can be attributed to the fact that the He···H_2_ system is weakly polarizable, and the total induction energy in this system is small and does not contribute much to the total interaction energy. The values obtained from the FF supermolecular CCSD(T) and FF-SAPT based on Hartree–Fock orbitals agree to within 17 and 8% for 5*a*_0_ and 7*a*_0_, respectively. At the same time, FF-SAPT(DFT) deviations reach 26 and 11%, respectively. In Table 2 and Figure 3, we can see that the polarization part is generally small in the valence-overlap region, whereas the exchange is large and is actually the dominant short-range contribution to the induced dipole moment. At long-range, however, the polarization effect quickly becomes dominant.

### 4.2 Nonpolar Molecules

**Figure 4:**
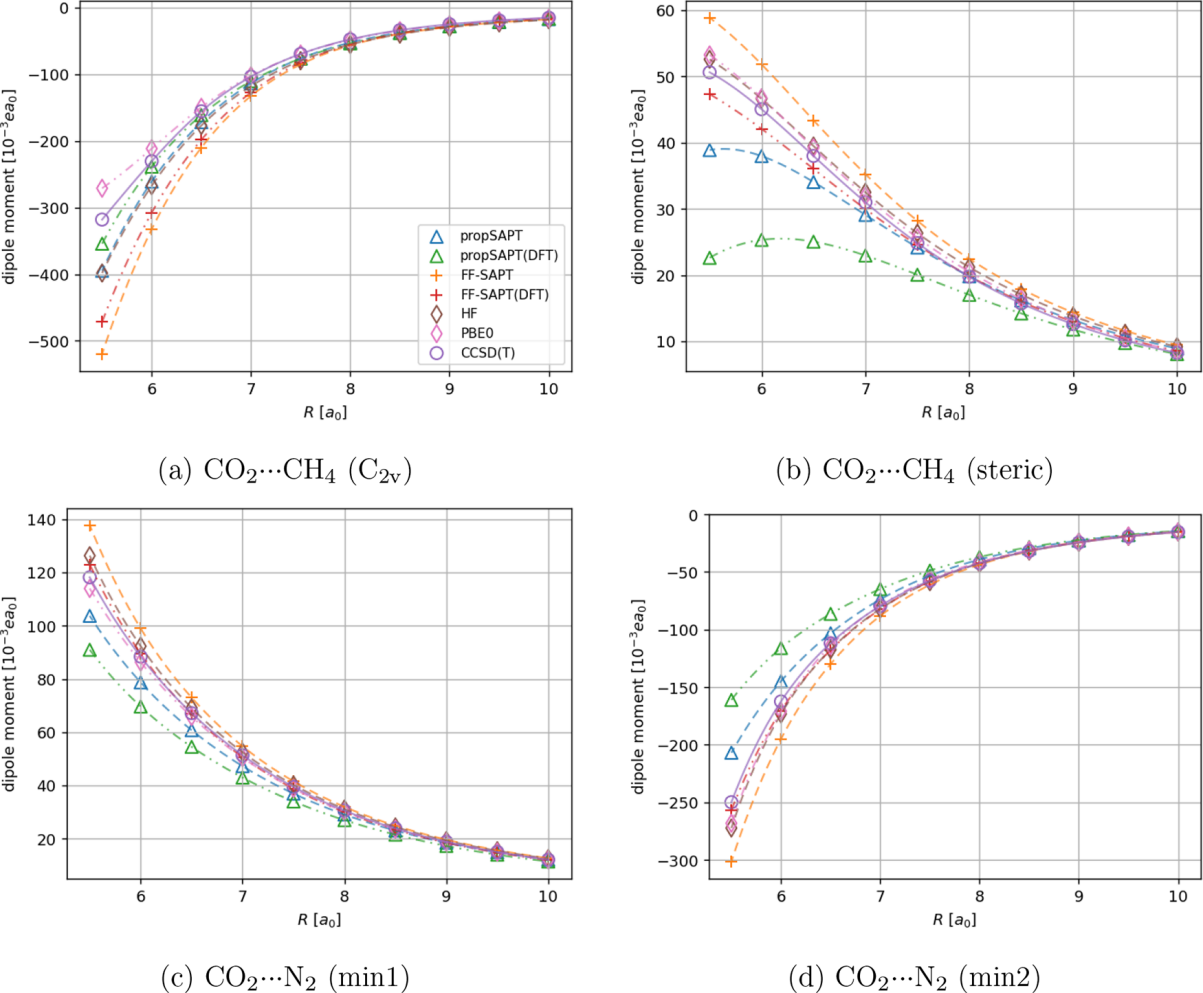
Distance-dependent comparison of interaction-induced dipole moments of the carbon dioxide bearing complexes calculated using the propSAPT approach, finite-field SAPT to the second order (FF-SAPT), and supermolecular methods. Panels a and b show results for the CO_2_···CH_4_ system in two orientations, while panels c and d pertain to the CO_2_···N_2_ complex. Note that the induced dipole moment vector is oriented along the intermolecular axis. Negative values indicate the induction of a dipole moment pointing toward CO_2_, positive values indicate the opposite direction (induced dipole moment pointing toward CH_4_ or N_2_). All values were obtained along radial cuts of the potential energy surface (PES) where the energy minima occur. See Figure 1 for the reference.

**Figure 5:**
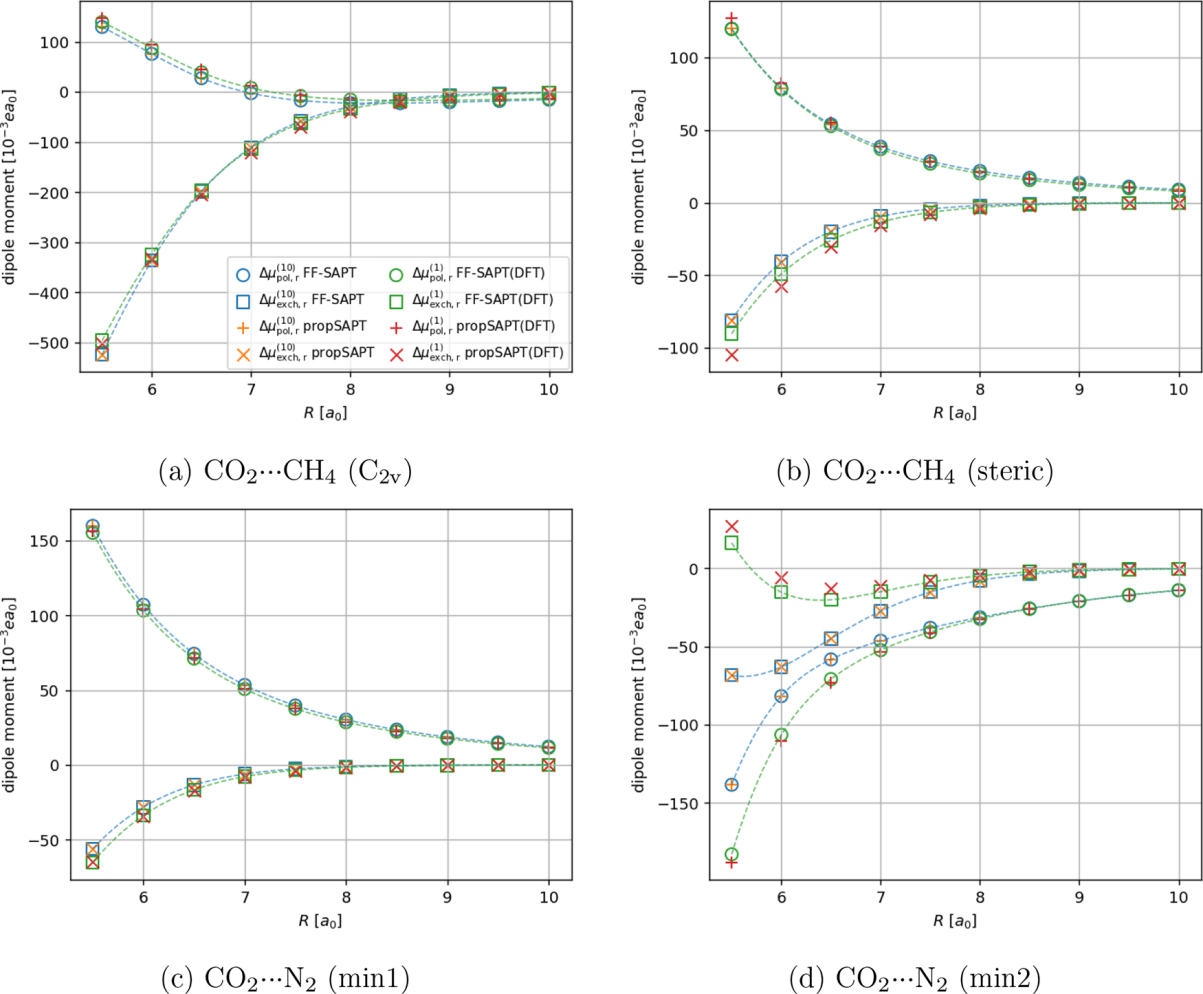
Distance-dependent comparison of the first-order polarization and exchange contributions to the interaction-induced dipole moment for the CO_2_ containing complexes. Note that for the Hartree–Fock-based propSAPT contributions (orange plus and cross markers), we expect an exact match with the finite-field numerical derivatives of SAPT (blue box and circle markers). Note that the induced dipole moment vector is oriented along the intermolecular axis. Negative values indicate the induction of a component pointing toward CO_2_, positive values indicate the opposite direction (pointing toward CH_4_ or N_2_).

**Figure 6:**
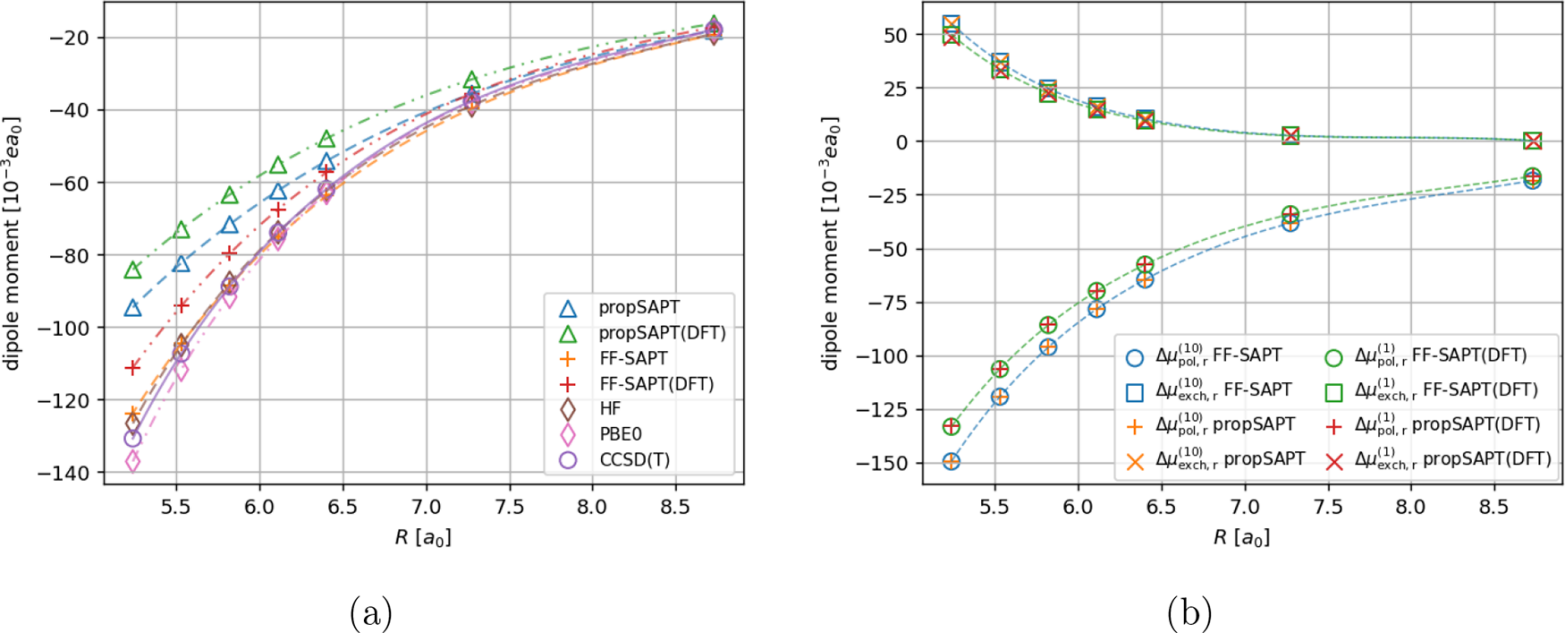
Distance-dependent comparison of interaction-induced dipole moment (panel a) and its first-order polarization and exchange contributions (panel b) for the Ar···BF_3_ complex. Note that the induced dipole moment vector is oriented along the intermolecular axis. Negative values indicate the induction of a dipole moment pointing toward Ar, positive values indicate the opposite direction (induced dipole moment pointing toward BF_3_).

For more complex nonpolar molecular systems, namely the CO_2_···N_2_, CO_2_···CH_4_ (see Figures 4 and 5), and Ar···BF_3_ (see Figure 6) cases, the propSAPT theory performs on average better than in the He···H_2_ case, with the deviations from CCSD(T) ranging between 0.2 and 28%, with a mean unsigned relative error of 9.7% for the Hartree–Fock orbitals. However, the maximum error is reduced to 14% at larger intermolecular separations. The Kohn–Sham description performs similarly well at long-range, but, quite surprisingly, it is somewhat more problematic at short-range. The maximum error occurs for the ‘steric’ configuration of CO_2_···CH_4_ (see Figure 4b), where the propSAPT(DFT) dipole moment is underestimated by 55%.

The overall good performance of propSAPT compared to the CCSD(T) reference in these systems can be attributed to the more significant contribution of electrostatic energy and the larger polarizability of the investigated species. If the electrostatic interaction dominates, the first-order contributions to the induced dipole are bigger; however, when dispersion interactions are more substantial, higher-order contributions become more critical for the qualitative reproduction of a physically correct picture.

### 4.3 Atom···Atom Systems

Our atom···atom test systems are different from the others since they are the only ones in which the long-range contribution of the second order is essential. These systems exhibit the asymptotic behavior of the interaction induced dipole moment as *R*^–7^, which would originate in the second order of the theory, not yet present in our formalism. However, it is good to test these systems to determine the importance of the contribution of dispersion and high-order polarization (induction) effects.

**Figure 7:**
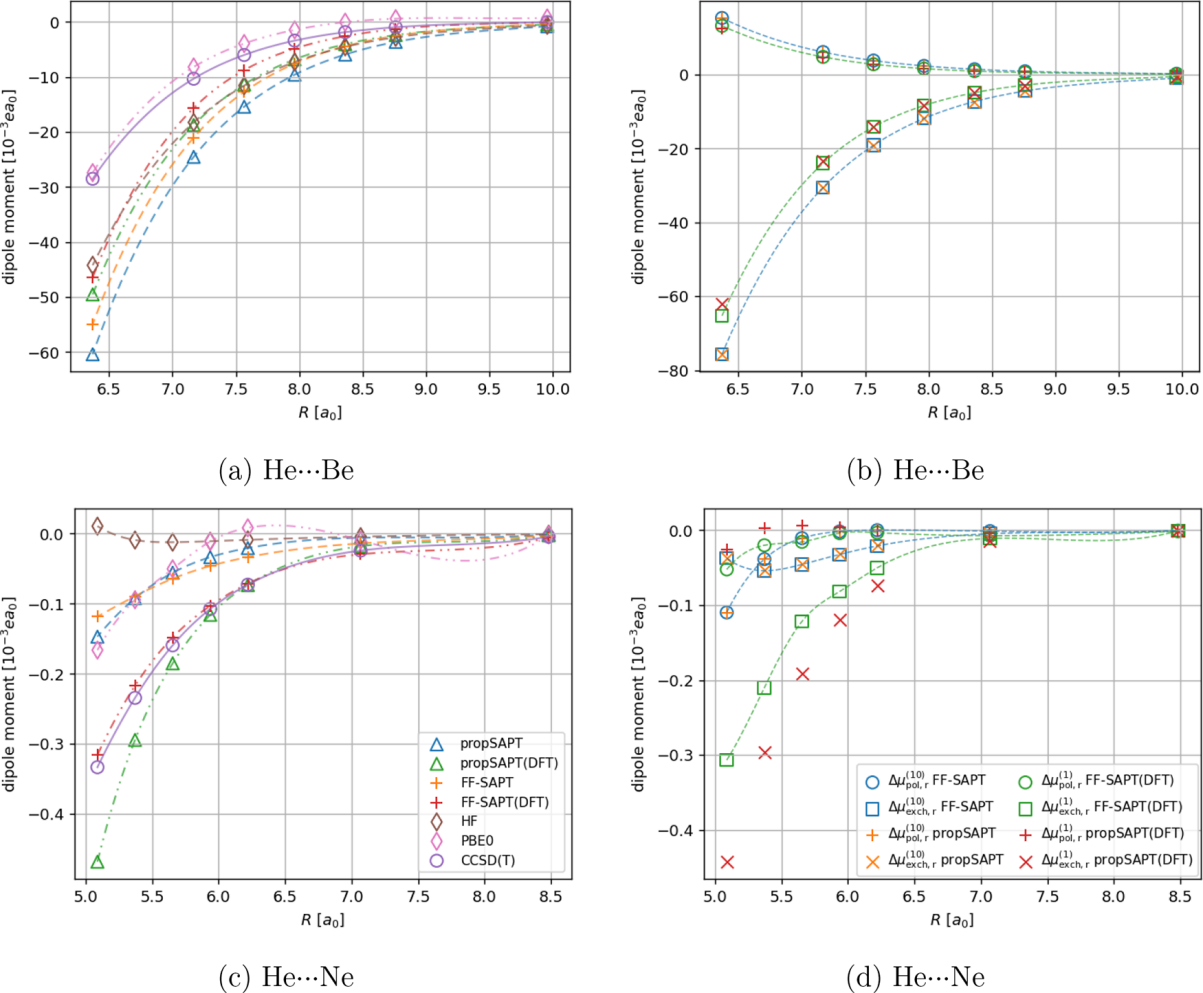
Distance-dependent comparison of interaction-induced dipole moments (panels a and c) and its first-order polarization and exchange contributions (panels b and d) for the He···Be and He···Ne systems. Note that the induced dipole moment vector is oriented along the intermolecular axis. Negative values indicate the induction of a dipole moment pointing toward He, positive values indicate the opposite direction (induced dipole moment pointing toward Be or Ne).

In Figure 7 the results for He···Be and He···Ne are visualized. The values obtained from the propSAPT method for the diatomic cases are lacking in accuracy compared to the finite-field CCSD(T) reference calculations, leading to the most significant unsigned relative errors observed among our test systems. This is partially likely due to the reason described in the above paragraph, i.e., not accounting for the dispersion effects crucial for the correct description of interactions between two closed-shell atoms. Surprisingly, for the He···Be case, both FF-SAPT and FF-SAPT(DFT) approaches are not constituting a notable improvement over their first-order propSAPT counterparts. The inclusion of second-order induction and dispersion effects does not lead to a significant improvement for this particular system. The He···Ne interaction, however, improves significantly when the second-order corrections are included but only for the Kohn–Sham description of monomers, i.e., in the FF-SAPT(DFT) case. For the diatomic cases studied in this work, the DFT versions of both propSAPT and finite-field SAPT methods perform better than the Hartree–Fock-based variants.

### 4.4 Polar Systems

**Figure 8:**
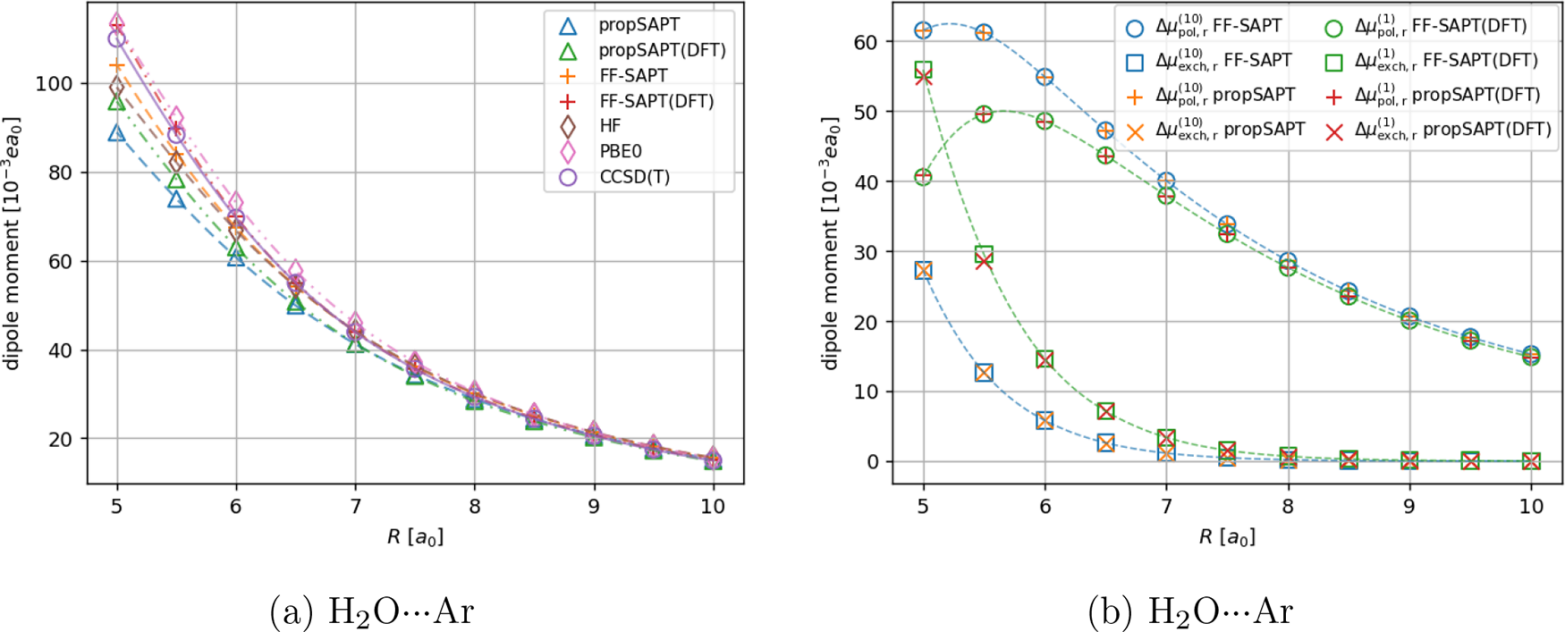
Distance-dependent comparison of interaction-induced dipole moment (panel a) and its first-order polarization and exchange contributions (panel b) for the H_2_O···Ar system. Note that for the induced dipole moment vector is oriented along the intermolecular axis. Negative values indicate the induction of a dipole moment pointing toward H_2_O, positive values indicate the opposite direction (induced dipole moment pointing toward Ar).

**Figure 9:**
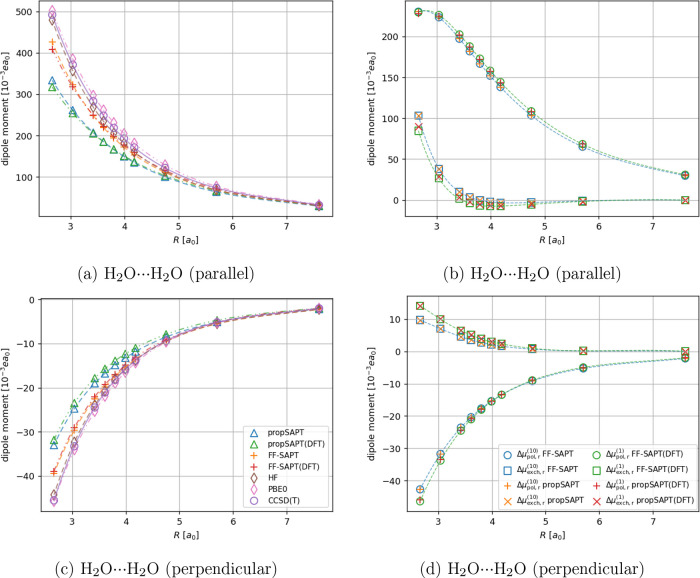
Distance-dependent comparison of interaction-induced dipole moments (panels a and c) and first-order polarization and exchange contributions (panels b and d) for the water dimer. For the parallel component the positive values indicate a component pointing toward the acceptor water molecule.

The distance-dependent comparison of the interaction-induced dipole moments and their first-order corrections for the test systems containing a polar molecule are presented in Figure 8 for the H_2_O···Ar complex and in Figure 9 for the water dimer, respectively.

In the case of the H_2_O···Ar complex, the Hartree–Fock-based version of propSAPT leads to at most 19% error compared to the CCSD(T) reference, whereas its Kohn–Sham-based variant performs even better with a maximum unsigned relative error of 13%. The performance of the FF-SAPT calculations for this system is very good, especially for the DFT-based approach, where the errors with respect to CCSD(T) were below 3%. For the Hartree–Fock version of the FF-SAPT, the agreement was observed to be within 6%.

Compared to the H_2_O···Ar complex, the performance of the tested methods in the water dimer case is slightly worse. The propSAPT method values of the induced dipole moment lead to at most 32 and 35% error for the theory’s Hartree–Fock- and DFT-based flavors, respectively. However, values of the induced dipole moment calculated with the FF-SAPT approaches are no worse than within 13% of unsigned error for the Hartree–Fock reference and within 17% for the DFT reference, compared to finite-field CCSD(T).

### 4.5 General Remarks

Interestingly, the directions of the polarization and exchange contributions in propSAPT can vary with respect to each other. Both polarization and exchange contributions may be pointing in the same direction simultaneously, as is observed for the (min2) configuration of the CO···N_2_ complex, or in the opposite directions like in Ar···BF_3_. Furthermore, these contributions also vary significantly in magnitude. This large variability results from the high sensitivity of charge distribution within the complex to various effects. Electrostatic (polarization) contributions can be of arbitrary sign (direction), depending on the donor or acceptor character of the atoms within the complex. Similarly, the Pauli exclusion principle can influence the exchange contribution in either direction, depending on the charge distribution within the molecule. This decomposition into the physically interpretable components opens up the possibility of analysis of the origin of charge transfer in noncovalently bound complexes, in an alternative way to those presented e.g. in refs (86 and 87). Furthermore, it should be mentioned that, in general, the first-order components of the interaction-induced dipole are not monotonous with respect to the intermolecular separation *R*, similar to what is observed for the total induced dipole.^88^

As observed in our test cases, the FF-SAPT and FF-SAPT(DFT) methods perform very well for more complex systems studied, agreeing on average within 22 and 13% with supermolecular finite-field CCSD(T) results, respectively. This demonstrates that FF-SAPT(DFT) can be effectively applied to dipole moment surfaces and may serve as a less computationally demanding alternative to expensive coupled-cluster calculations, additionally providing valuable insights into the type of interaction driving the property changes.

For some test systems studied in this work, the discrepancies between the values of first-order induced dipole moment corrections from propSAPT(DFT) and FF-SAPT(DFT) were observed. These are mainly due to the ALDA approximation of the exchange-correlation kernel used in CPKS calculations, being a part of the propSAPT(DFT) routine. The discrepancies were well visible especially for the CO_2_···CH_4_ in the ‘steric’ configuration (see Figure 5a), CO_2_···N_2_ in the orientation corresponding to min2 (Figure 5d) and He···Ne (Figure 7d). It was also observed that the usage of the ALDA kernel for the response had a significantly worse impact on the first-order exchange correction compared to its polarization counterpart.

## 5 Conclusions and Outlook

In this paper, we have introduced a new formalism for computing first-order properties within the SAPT framework. Such a formalism facilitates the perturbative and direct calculation of changes in one-electron properties without the necessity of solving the Schrödinger equation for the dimer. The formalism is based on the previously introduced method of direct calculation of interaction-induced properties by Piszczatowski et al.^50^; however, we have integrated it with SAPT by approximating the dimer wave function using the antisymmetrized product of monomer determinants. This approach leads to closed-form expressions, allowing any one-electron property to be expressed exclusively in terms of monomer orbitals. We refer to this method as propSAPT. In this initial development, we derived, implemented, and evaluated the propSAPT expressions to first order in the intermolecular interaction operator.

In order to test our theory, we compared the interaction-induced dipole moments from propSAPT with finite-field supermolecular calculations and finite-field SAPT based on both Hartree–Fock (FF-SAPT) and DFT description of monomers (FF-SAPT(DFT)) for several systems, namely: He···H_2_, Ar···BF_3_, CO_2_···N_2_, CO_2_···CH_4_, He···Be, He···Ne, H_2_O···Ar and H_2_O···H_2_O. Since our expressions for the SAPT correction derivatives obey the Hellman-Feynman theorem, we could test their performance rigorously by comparing them with a dipole moment obtained by differentiation of the first-order SAPT energies with respect to the electric field strength. The Hartree–Fock-based propSAPT first-order corrections calculated using analytical derivatives agreed in the worst case within 1.18 × 10^–5^*ea*_0_ with the corresponding numerical derivatives, assuring us of the correctness of the implementation of the newly derived theory. Given the agreement with the finite-field approach, one can conclude that our theory correctly accounts for the orbital relaxation. Overall, the first-order propSAPT theory based on Hartree–Fock performs quite well for the induced dipole moments. Excluding the atom···atom cases, it is leading on average to 28% error with respect to finite-field CCSD(T). Regarding the Kohn–Sham-based propSAPT(DFT) equivalent, using the PBE0 functional leads to 35% average error (atom···atom cases excluded) compared to reference finite-field CCSD(T) calculations. For molecular systems dominated by electrostatic interactions, the performance of propSAPT is even better. For the long-range region of the dipole moment, the first-order propSAPT agrees very well with CCSD(T). Note that the DFT formalism presented here can be combined with methods that are based on ab initio electron densities,^89,90^ similar to what was recently demonstrated by Boese and Jansen.^91^

Based on the numerical tests gathered, the performance of finite-field SAPT approaches leads on average to 28% error for HF-based and 13% error for DFT-based calculations with respect to the CCSD(T) reference. Considering the favorable computational scaling of the FF-SAPT(DFT) method, it may constitute an insightful alternative to the supermolecular approach for dipole moment surface calculations for many-electron systems.

The presented theory may find applications in the calculations of interaction-induced property surfaces for complex systems, such as the dipole moment surface (DMS) for polyatomic molecules. Given the high cost and complexity of DMS calculations, a method that primarily involves monomer wave functions presents a significant advantage. Examples of such systems could include the DMS of molecules in aqueous solutions or the collision-induced absorption coefficient (CIA) for systems like methane–methane or methane-carbon dioxide. Recently, interest in the calculation of DMS has increased enormously due to their critical role in spectroscopy. High-resolution spectroscopic observations, when combined with modern astronomical instruments such as the James Webb Space Telescope (JWST), have the potential to revolutionize our understanding of planet formation and the search for biosignatures.^1,92^ Notably, the first CIA feature was recently observed by JWST.^93^

An additional benefit of propSAPT is the insight gained from decomposing the induced dipole moment into distinct contributions: polarization- and exchange-driven. This significantly enhances the interpretative power of SAPT, enabling the quantification and analysis of effects such as the origin of charge transfer and delocalization through the examination of dipole moment changes and their physical sources. It should be noted that the presented theory can also provide insights into interaction-induced changes in density matrices. Naturally, Δ*X* can be considered as the trace of the property operator *X* with a change in the density matrix. This matrix can reveal information on the density changes resulting from molecular interactions and their decomposition into physically well-defined contributions in the spirit of SAPT. Given the interpretive power of such an approach, one can relate density changes to specific physical effects. Finally, the theory outlined here can be adapted to include geometric analytical derivatives for SAPT corrections, opening the door to many other applications such as SAPT-based geometry optimizations or direct calculations of frequency shifts in van der Waals complexes. Work on these extensions of the presented theory actively continues in our groups.

## Supporting Information Available

Contents of Supporting Information files (PDF)
Contains .xyz files with the molecular geometries studied (ZIP)
All calculated values of the interaction-induced dipole moments (XLSX)
