# Supplementary material for: Analytical Derivatives of Symmetry-Adapted Perturbation Theory Corrections for Interaction-Induced Properties
Source: J Chem Theory Comput. 2025 Apr 25;21(9):4562–78. doi: 10.1021/acs.jctc.5c00238 (PMC12079802; doi:10.1021/acs.jctc.5c00238)
Supplement: Supplementary file 3 — ct5c00238_si_003.pdf [file ct5c00238_si_003.pdf]

Supporting information for the article: Analytical derivatives of symmetry-adapted perturbation theory corrections for interaction-induced properties

Bartosz Tyrcha,[*,†] Tarun Gupta,[†] Konrad Patkowski,[*,‡] and Piotr S. Żuchowski [*,†]

†*Institute of Physics, Faculty of Physics, Astronomy and Informatics, Nicolaus Copernicus University in Toruń, Grudziądzka 5/7, 87-100 Toruń, Poland*

‡*Department of Chemistry and Biochemistry, Auburn University, Auburn, Alabama 36849, USA*

E-mail: btyrcha@doktorant.umk.pl; patkowsk@auburn.edu; pzuch@fizyka.umk.pl

Contents

1 Results **2**

2 Geometries **3**

1 Results

The .xlsx spreadsheet file contains computational results for tested molecular systems, including interaction-induced dipole moments. The columns are:

- System: molecular system studied,
- R: intermolecular separation (in a_0),
- Theta: angle parameter (in degrees),
- Component: vector component of interaction-induced dipole moment,
- Nuclear: nuclear dipole moment of a dimer,
- propSAPT X1_pol,r, propSAPT X1_exch,r, propSAPT total: dipole moment from propSAPT,
- FF-SAPT X1_pol, FF-SAPT X1_exch, FF-SAPT X2_ind, FF-SAPT X2_exch-ind, FF-SAPT X2_disp, FF-SAPT X2_exch-disp, FF-SAPT total: dipole moment from FF-SAPT,
- propSAPT(DFT) X1_pol,r, propSAPT(DFT) X1_exch,r, propSAPT(DFT) total: dipole moment from propSAPT(DFT),
- FF-SAPT(DFT) X1_pol, FF-SAPT(DFT) X1_exch, FF-SAPT(DFT) X2_ind, FF-SAPT(DFT) X2_exch-ind, FF-SAPT(DFT) X2_disp, FF-SAPT(DFT) X2_exch-disp, FF-SAPT(DFT) total: dipole moment from FF-SAPT(DFT),
- HF: dipole moment from supermolecular HF,
- PBE0: dipole moment from supermolecular DFT (PBE0 functional),
- CCSD(T): dipole moment from finite-field supermolecular CCSD(T).

2 Geometries

The `.zip` archive contains `.xyz` files with the molecular geometries studied. The filenames are indicating the molecular system, the intermolecular separation (in a_0) and, in the case of $He \cdots H_2$ complex, also the angle parameter (in degrees).
